# Improving the EHMRG Prognostic Evaluation of Acute Heart Failure with TAPSE/PASp: A Sequential Approach

**DOI:** 10.3390/diagnostics12020478

**Published:** 2022-02-13

**Authors:** Lorenzo Falsetti, Vincenzo Zaccone, Giovanna Viticchi, Agnese Fioranelli, Ilaria Diblasi, Emanuele Guerrieri, Consuelo Ferrini, Mattia Scarponi, Luca Giuliani, Caterina Scalpelli, Marianna Martino, Adolfo Pansoni, Marinella Luccarini, Maurizio Burattini, Gianluca Moroncini, Nicola Tarquinio

**Affiliations:** 1 Internal and Subintensive Medicine Department, Azienda Ospedaliero-Universitaria “Ospedali Riuniti”, 60126 Ancona, Italy; vincenzozaccone@libero.it; 2 Neurologic Clinic, Università Politecnica delle Marche, 60126 Ancona, Italy; viticchi.giovanna@ospedaliriuniti.marche.it or; 3 Internal Medicine Department, INRCA-IRCCS di Osimo (Ancona), 60027 Ancona, Italy; agnese.fioranelli@gmail.com (A.F.); caterina.scalpelli@gmail.com (C.S.); mariannamartino88@gmail.com (M.M.); m.luccarini@inrca.it (M.L.); m.burattini@inrca.it (M.B.); n.tarquinio@inrca.it (N.T.); 4 Emergency Medicine Residency Program, Università Politecnica delle Marche, 60126 Ancona, Italy; ilariadiblasi@gmail.com (I.D.); e.guerrieri93@gmail.com (E.G.); consueloferrini@hotmail.it (C.F.); mattia.scarponi93@gmail.com (M.S.); luca.giuliani33@gmail.com (L.G.); 5 Emergency Department, INRCA-IRCSS Osimo, 60027 Ancona, Italy; a.pansoni@inrca.it; 6 Clinica Medica, Università Politecnica delle Marche, 60126 Ancona, Italy; g.moroncini@univpm.it

**Keywords:** acute heart failure, echocardiography, TAPSE/PASp, EHMRG score, clinical score, prognosis

## Abstract

The Emergency Heart Failure Mortality Risk Grade (EHMRG) can predict short-term mortality in patients admitted for acute heart failure (AHF) in the emergency department (ED). This paper aimed to evaluate if TAPSE/PASp, an echocardiographic marker of ventricular desynchronization, can improve in-hospital death prediction in patients at moderate-to-high risk, according to EHMRG score classification. From 1 January 2018 to 30 December 2019, we retrospectively enrolled all the consecutive subjects admitted to our Internal Medicine Department for AHF from the ED. We performed bedside echocardiography within the first 24 h of admission. We evaluated EHMRG and NYHA in the ED, days of admission in Internal Medicine, and in-hospital mortality. We assessed cutoffs with ROC curve analysis and survival with Kaplan–Meier and Cox regression. We obtained a cohort of 439 subjects; 10.3% underwent in-hospital death. Patients with normal TAPSE/PASp in EHMRG Classes 4, 5a, and 5b had higher survival rates (100%, 100%, and 94.3%, respectively), while subjects with pathologic TAPSE/PASp had lower survival rates (81.8%, 78.3%, and 43.4%, respectively) (*p* < 0.0001, log-rank test). TAPSE/PASp, an echocardiographic marker of ventricular desynchronization, can further stratify the risk of in-hospital death evaluated by EHMRG.

## 1. Introduction

Heart failure (HF) is a global public health problem characterized by high mortality and increased hospitalization rates [1]. Acute heart failure (AHF) is often managed first in the emergency department (ED); approximately one third of AHF patients are discharged directly or managed in short-stay [2], while others are admitted to cardiology or internal medicine departments. Patients discharged directly from the ED have the highest risk of short-term adverse outcomes [3]; these subjects show high mortality, increased re-hospitalization rates leading to lower quality of life, and increased healthcare costs [4]. AHF may present with heterogeneous symptoms and several degrees of severity that do not necessarily correlate with prognosis. In fact, patients with more severe clinical symptoms may have a more favorable prognosis than patients with milder presentations [5,6]. Since there is no consistent clinical-prognostic association, adopting only clinical judgment in the ED leads to over-hospitalization of low-risk patients, while the higher-risk ones could be inappropriately discharged [7]. Therefore, formal risk stratification is necessary to optimize patients’ management, adopting specific scores that can be applied before making decisions about patients’ disposition [3]. Currently, there are many clinical scores to stratify AHF prognosis, but most of them have been validated only in specific settings, and their generalization should be cautious especially in Emergency Departments, Intensive Care Units (ICU), and Internal Medicine departments. In fact, several robust and validated scores shown low accuracy in predicting short-term mortality in specific settings, as in the ICU [8] or ED [9]. Moreover, regional variations of risk factors and comorbidities, mainly due to different standards of living, life expectancy, quality of care, and efficacy of sanitary systems, can account for a substantial variation of accuracy of predictive scores in different populations [10]. Several predictive systems have been designed and validated specifically for ED use, such as EHMRG [11], OHFRS [12], MEESSI [13], and AHFRS [9] scores, among others. The use of specific prognostic tools [14] could allow resource optimization and an ED physician’s disposition for rationalization. In particular, an accurate identification of low-risk subjects could be useful to identify subjects who could be safely discharged from ED or short-stay areas, since these patients show the highest risk of short-term complications [2]. For this study, we chose to adopt the EHMRG score, since it was specifically designed in the ED to assess short-term mortality for AHF [11], ensuring a patient’s risk stratification since their arrival. Moreover, it was validated in different populations with similar results [15,16]. We aimed to assess, in a population of elderly subjects admitted for AHF, whether a sequential approach based on an initial EHMRG evaluation, and a subsequent risk stratification based on an echocardiographic marker, namely TAPSE/PASp, was able to identify patients at lower risk of short-term mortality among subjects classified as in moderate-to-high risk according to EHMRG score classification.

## 2. Materials and Methods

In the timeframe between 1 January 2018 and 30 December 2019, we retrospectively assessed all the consecutive patients evaluated in the ED of INRCA-IRCSS Hospital (Osimo, Ancona, Italy) and subsequently admitted with an AHF diagnosis to the Internal Medicine department of the same hospital. The INRCA-IRCSS (National Institute for Care and Research in Ageing) Hospital of Osimo (Ancona, Italy) is fully dedicated to the care and research of geriatric diseases, and it is specialized in acute care of elderly patients. Regarding AHF, the ED of the structure has a direct access to the Internal Medicine department. Patients affected AHF associated to other acute illnesses, as for example acute coronary syndromes (both STE and NSTE) requiring urgent revascularization, arrhythmias requiring pacing, acute valvular diseases or cardiac diseases requiring cardiac surgery, or associated critical illnesses requiring intensive care unit admission (i.e., pulmonary embolism, septic or cardiogenic shock) are directly sent or to the INRCA cardiac care unit or to a larger tertiary-care hospital after pre-hospital or ED evaluation. Thus, “very-high risk” clinical conditions were not present in the enrolled cohort, which comprised mostly patients affected by acutely decompensated heart failure (ADHF) or de novo AHF due to medical conditions (arrhythmias, hypertensive crises, and other causes). The diagnosis of ADHF/AHF was given by the attending physician according to the ESC 2016 guidelines that were current at the moment of the study [17]. For each enrolled subject, we collected the following data: age, sex, days of admission, BNP values at admission (pg/mL), and NYHA class. At ED arrival, we calculated, for each subject, the EHMRG score by assessing the necessary items: arrival by ambulance, systolic blood pressure (SBP), heart rate (HR), oxygen saturation (SpO_2_), serum potassium levels, serum creatinine levels, serum troponin levels, presence of active cancer, and metolazone use. We then calculated the absolute EHMRG score, according to its original definition, as synthesized in Table 1; then we categorized the patients into the six EHMRG quintile categories (Class 1: −49.1; Class 2: from −49.0 to −15.9; Class 3: from −15.8 to 17.9; Class 4: from 18.0 to 56.5; Class 5a: from 56.6 to 89.3; Class 5b: 89.4) [11].

Within 24 h from hospital admission, patients were submitted to an extended bedside echocardiography evaluating eyeballing left ventricle ejection fraction (LVEF), classified as reduced and preserved, E/e’ ratio, E/A ratio, TAPSE (mm), PASp (mmHg), TAPSE/PASp ratio (mm/mmHg), and indexed left atrial volume (ml/m^2^). These measurements followed ASE guidelines for transthoracic cardiac ultrasound assessment [18].

### 2.1. Ethical Issues

This study was authorized in 06.05.2021 by the INRCA-IRCSS Ethical Committee (CE INRCA, protocol no 21011/21-CE), and then approved by INRCA Hospital (protocol n° 193, 26 May 2021). All patients gave their informed consent and were treated according to the guidelines current at the time of the study and to the Helsinki declaration and followed the Declaration of Helsinki Ethical Principles for Medical Research Involving Human Subjects. 

### 2.2. Inclusion and Exclusion Criteria

We included in the analysis all the subjects admitted to the Internal Medicine department from ED with a diagnosis of AHF, aged 60 or more years. We adopted the same exclusion criteria of the original EHMRG study [11]: (i) transfer from another department (ICU, Cardiology, Pneumology) or direct admission from the HF ward, (ii) patients on end-of-life care due to active cancer or other terminal comorbidities, (iii) dialysis-dependent subjects. We also excluded patients with incomplete data that did not allow us to calculate correctly the EHMRG score. We did not exclude patients without available echocardiographic data. However, if the exam was performed after the first 24 h from admission, we did not use the echocardiographic data.

### 2.3. Statistical Analysis

Anonymized data were collected in an electronic database. Continuous variables were tested for normality using the Kolmogorov–Smirnov test: normally distributed variables were presented as mean and standard deviation (SD) and compared with the *t*-test (two variables) or ANOVA test (multilevel variables). Non-normally distributed variables were presented as median and interquartile range [IQR] and compared with the non-parametric Mann–Whitney U-test (two variables) or Kruskal–Wallis H-test (multilevel variables). We considered and analyzed as continuous the following variables: age, sex, days of admission, BNP values on admission (pg/mL), BNP values on discharge (pg/mL), SBP, HR, SpO_2_, serum potassium, creatinine and troponin levels, absolute EHMRG score value, E/e’ ratio, E/A ratio, TAPSE (mm), PASp (mmHg), TAPSE/PASp ratio (mm/mmHg), and indexed left atrial volume (ml/m2). Categorical and dichotomous variables were presented as absolute numbers and percent and compared with the chi-square test. We treated as dichotomous or categorical variables: sex, arrival in the ED by ambulance, active cancer, home use of metolazone, and LVEF. We also coded as dichotomous (i) an abnormal troponin, defined as a troponin level above the 99th percentile, and (ii) an abnormal potassium, defined as an absolute potassium level ≤ 3.9 mmol/L or ≥4.6 mmol/L. Sensitivity (Se), specificity (Sp), and area under the curve (AUC) in predicting in-hospital mortality of specific continuous variables of interest (EHMRG, TAPSE/PASp, and BNP) were assessed by ROC curve analysis. The best cutoff point for each variable was selected by critical evaluation of ROC curves and by adopting the Youden Index. We then selected the best cutoff point and recoded each continuous variable into a dichotomous one. Survival analysis was performed using Kaplan–Meier curves, adopting the log-rank test for comparison. We selected the covariates included in the multivariate model using Pearson’s bivariate analysis. When the outcome variable and the covariate resulted associated at a <0.05 level, we included that variable in the final model. The full Cox regression model considered in-hospital mortality as the main outcome variable, days of hospitalization as the temporal variable, binary TAPSE/PASp as predictor, BNP on admission, and NYHA class as covariates, and the categorical EHMRG variable as a stratum variable. Statistical analysis was performed with MedCalc 10.0 (MedCalc Software) and SPSS 13.0 software (SPSS Inc., Chicago, IL, USA) for Windows systems.

## 3. Results

From an initial sample of 546 subjects, we excluded 26 patients for dialysis-dependent state, 32 for transfer from another department, and 36 for the coexistence of advanced oncologic pathologies requiring only end-of-life care. In total, 13 patients had incomplete clinical and laboratoristic data, thus they were excluded from analysis. Echocardiographic data obtained within the first 24 h were available for a sub-sample of 289 subjects. We obtained 439 consecutive patients, with a mean age of 84.6 (±7.7) years, and males representing 41.0% of the cohort. Within admission, we observed 45 in-hospital deaths, representing 10.3% of the sample. We also collected the clinical, laboratoristic, and echocardiographic characteristics and the cause of admission [ADHF or de novo AHF] of the considered subjects, and summarized the baseline characteristics of the sample in Table 2. Of note, the characteristics of our cohort and the explored outcomes are similar to the original and to the validation cohort, except for age, which is obviously higher among our subjects [11,19].

AHF de novo (n, %)

**Figure 1:**
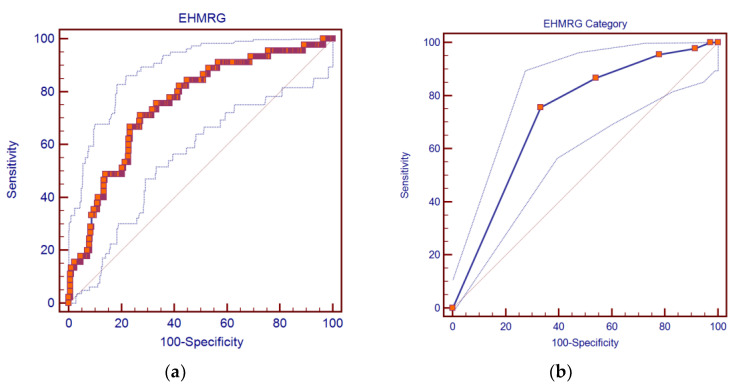
ROC curves of: (**a**) EHMRG score, considered as a continuous variable (AUC: 0.753; 95%CI: 0.710–0.793; *p* < 0.0001); (**b**) EHMRG score, considered as a categorical variable (AUC: 0.727; 95%CI: 0.683–0.768; *p* < 0.0001).

The EHMRG score was fairly accurate in predicting in-hospital death (AUC: 0.753; 95%CI: 0.710–0.793; *p* < 0.0001), as shown in Figure 1a. We observed a similar performance when treating EHMRG as a categorical variable (AUC: 0.727; 95%CI: 0.683–0.768; *p* < 0.0001), as shown in Figure 1b. 

Analyzing the values of this second curve, we observed that the EHMRG score had an optimal cutoff at category 3 in this sample. At this point, we obtained a sensitivity of 95.56% (95%CI: 84.9–99.5%), a specificity of 22.08% (95%CI: 18.1–26.5%), a positive likelihood ratio of 1.23 (95%CI: 1.1–1.3), a negative likelihood ratio of 0.20 (95%CI: 0.05–0.8), a positive predictive value of 12.3% (95%CI: 9.0–16.2%), and a negative predictive value of 97.8% (95%CI: 92.1–99.7%). 

**Figure 2:**
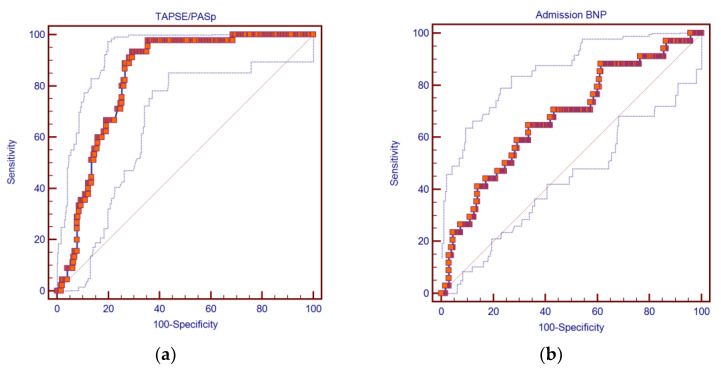
ROC curves of: (**a**) TAPSE/PASp, considered as a continuous variable (AUC: 0.831; 95%CI: 0.782–0.872; *p* < 0.0001); (**b**) admission BNP, considered as a continuous variable (AUC: 0.667; 95%CI: 0.624–0.706; *p* < 0.0001).

Of the echocardiographic indicators, TAPSE/PASp proved to be a useful index to predict in-hospital mortality (AUC: 0.831; 95%CI: 0.782–0.872; *p* < 0.0001), as shown in Figure 2a. TAPSE/PAPs had a sensitivity of 93.33% (95%CI: 81.7–98.6%), a specificity of 70.6% (95%CI: 64.5–76.2%), a positive likelihood ratio of 3.18 (95%CI: 2.6–3.9), a negative likelihood ratio of 0.094 (95%CI: 0.03–0.3), a positive predictive value of 36.8% (95%CI: 28.0–46.4%), and a negative predictive value of 98.3% (95%CI: 95.1–99.6%). 

BNP at admission resulted as a useful marker of prediction for in-hospital mortality, even if it performed less efficiently than EHMRG or TAPSE/PASp (AUC: 0.667; 95%CI: 0.624–0.706; *p* < 0.0001), as shown in Figure 2b. This yielded a sensitivity of 62.86% (95%CI: 44.9–78.5%), a specificity of 67.00% (95%CI: 62.7–71.1%), a positive likelihood ratio of 1.90 (95%CI: 1.40–2.50), a negative likelihood ratio of 0.55 (95%CI: 0.4–0.9), a positive predictive value of 11.9% (95%CI: 7.6–17.5%), and a negative predictive value of 96.2% (95%CI: 93.6–98%). 

**Figure 3:**
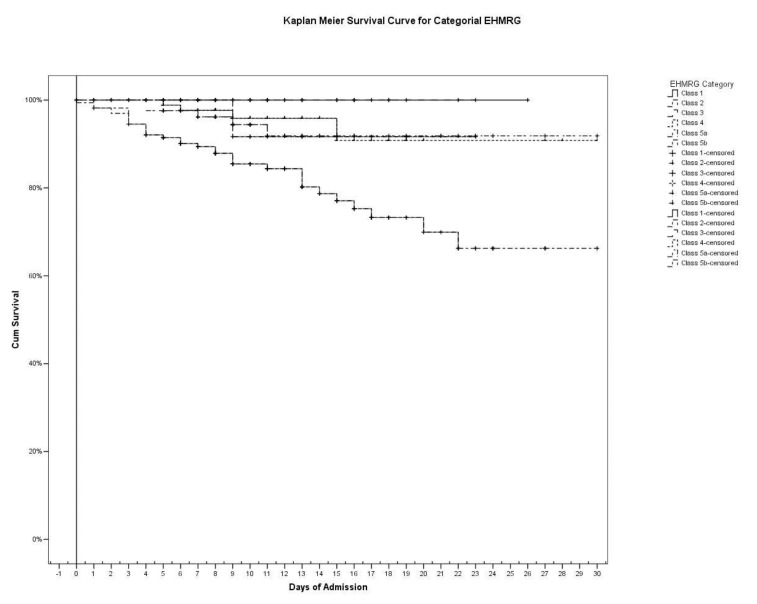
Kaplan–Meier curve of EHMRG, considered as a categorial variable (*p* = 0.0001, log-rank test).

**Figure 4:**
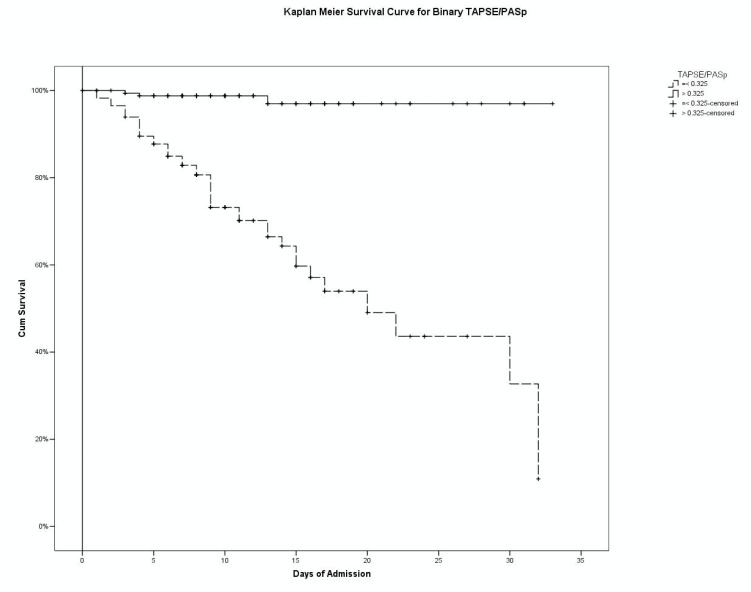
Kaplan–Meier curve of TAPSE/PASp, considered as a dichotomous variable (*p* = 0.0001, log-rank test).

The Kaplan–Meier curves showed that—in the analyzed cohort—both the categorical EHMRG score and the binary TAPSE/PASp were significantly associated to a different in-hospital mortality (Figure 3 and Figure 4). Considering EHMRG, Kaplan–Meier curves analysis showed that 100% of patients in Class 1 survived hospital admission, and that this percentage progressively decreased as the class increased (95.0% in Class 2; 97.2% in Class 3, 93.8% in Class 4, 90.7% in Class 5a, 68.9% in Class 5b, *p* < 0.0001, log-rank test). According to this analysis, 97.6% of patients with normal TAPSE/PASp did not experience death during hospitalization, while only 64.6% of subjects with pathological TAPSE/PASp survived to the acute event (*p* < 0.0001 at log-rank test). 

**Figure 5:**
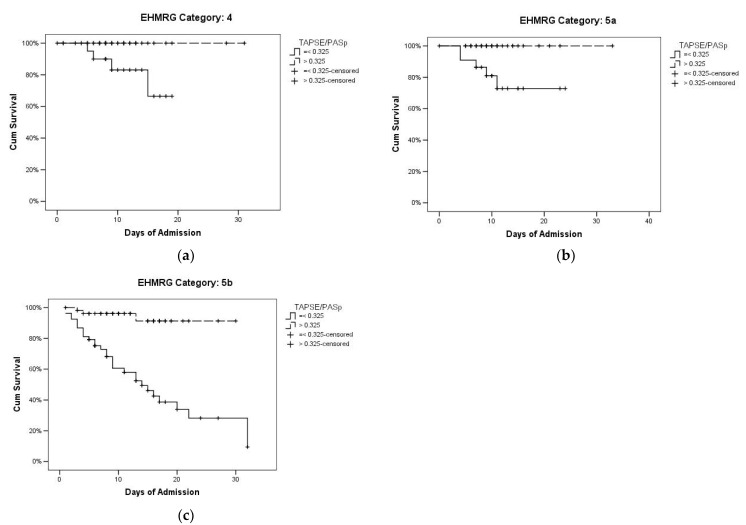
Kaplan–Meier’s curves of survival for TAPSE/PASp in (**a**) EHMRG category 4 (*p* = 0.010, log-rank test); (**b**) EHMRG category 5a (*p* = 0.010, log-rank test), (**c**) EHMRG category 5b (*p* < 0.0001, log-rank test).

Considering the impact of an additional stratification approach, we observed that a pathological TAPSE/PASp further stratified AHF prognosis in the advanced EHMRG classes, as Class 4 (Figure 5a), Class 5a (Figure 5b) and Class 5b (Figure 5c). In EHMRG class 4, a normal TAPSE/PASp was associated with 100% survival, while a pathological TAPSE/PASp was associated with 81.8% survival (Figure 5a, *p* = 0.010 at log-rank test). In EHMRG Class 5a, a normal TAPSE/PASp was associated with 100% survival, while a pathological TAPSE/PASp was associated with 78.3% survival (Figure 5b, *p* = 0.010 at log-rank test). In EHMRG Class 5b, a normal TAPSE/PASp was associated with 94.3% survival, while a pathological TAPSE/PASp was associated with 43.4% survival (Figure 5c, *p* < 0.0001 at log-rank test). This stratification, however, was of little use in the low-risk classes (1 to 3), given the low number of events observed (two patients out of 89 in EHMRG categories 1–3) and the close to 100% survival. 

Pearson’s bivariate test underlined that in-hospital death was associated with admission BNP (correlation = 0.143; *p* = 0.008), NYHA class (correlation = 0.265; *p* = 0.0001), age (correlation = 0.108; *p* = 0.024), ambulance arrival (correlation = 0.108; *p* = 0.023), SBP in the ED (correlation = 0.188; *p* = 0.0001), serum creatinine levels in the ED (correlation = 0.248; *p* = 0.0001); serum potassium (correlation = 0.103; *p* = 0.030), increased troponin in the ED (correlation = 0.212, *p* = 0.0001), active cancer (correlation = 0.140; *p* = 0.003), EHMRG (correlation = 0.292; *p* = 0.0001), TAPSE/PASp (correlation = 0.348; *p* = 0.0001), dichotomous LVEF (correlation = 0.122; *p* = 0.038), left atrium volume (correlation = 0.195; *p* = 0.007), EHMRG categories (correlation = 0.227; *p* = 0.0001), and dichotomous TAPSE/PASp (correlation = 0.459; *p* = 0.0001).

According to Pearson’s bivariate analysis results, we identified the following factors as associated with in-hospital mortality at a level of *p* < 0.05, thus to be included in the multivariate Cox test: BNP, NYHA class, categorical EHMRG, dichotomous TAPSE/PASp, dichotomous LVEF, and left atrium volume. All of those factors associated with mortality (age, serum potassium, serum troponin, metolazone use, active cancer, and ambulance arrival) contained in the EHMRG score or the TAPSE/PASp were eliminated from the multivariate test to reduce multicollinearity in the model. 

The Cox analysis, carried out considering the days of hospitalization as a temporal variable, in-hospital mortality as an event variable, and categorial EHMRG, binary TAPSE/PASp, BNP on admission, NYHA class, LVEF, and left atrium volume as predictors, showed the results shown in Table 3. A one-point increase in EHMRG category resulted in a 2.21-fold increase in the hazard ratio (HR) of in-hospital death. Similarly, a one-point increase in NYHA class was associated with a 2.99-fold increase in HR. It should be noted that the presence of pathological TAPSE/PASp is associated with an approximately 37.9-fold HR increase, whereas BNP and LVEF lost their predictive power in the presence of the other variables. Small increases on LA volume were associated to small but significant increases in the HR of in-hospital death, as shown in Table 3.

Performing multivariate Cox analysis in EHMRG Classes 4, 5a, and 5b, maintaining the same covariates and TAPSE/PASp as the main predictor, we observed substantial stability in HRs and underlined the same survival distribution observed in the Kaplan–Meier curves, suggesting the same reclassifying ability of TAPSE/PASp only considering the more advanced EHMRG classes.

## 4. Discussion

AHF represents a major health problem, especially among elderly patients [4]. In fact, it is characterized by both a high mortality and a high risk of early rehospitalization, especially among patients discharged directly from the ED [2]. However, there are no objective measures for disposition decision making, which is crucial for resource utilization. Particularly, there is no evidence-based system to assess subjects who will benefit of short-term or observational stays. Moreover, ED physician’s clinical gestalt is not accurate in predicting prognosis, and it seems ineffective in identifying patients at highest risk of adverse events or even death after a discharge disposition [5,6]. Appropriately designed clinical and laboratoristic scores, such as EHMRG, OHFRS, and MEESSI, are helpful in guiding the ED physician to correctly discharge or admit the patient [20]. However, both the MEESSI and OHFRS scores [12,13] require specific laboratoristic exams or instrumental evaluations, such as NT-pro-BNP and electrocardiographic modifications, to improve their prediction. The original EHMRG score does not use natriuretic peptides or electrocardiographic alterations to improve its performances, as shown in Table 1 [11]. This score is not time-expensive for the ED physician, who can calculate it from triage data and common laboratory exams with an easy calculation or several free smartphone apps. Moreover, cardiac and lung ultrasound is suggested at the admission of the patient with AHF to perform a differential diagnosis and to improve the AHF diagnosis itself [1,17]. Thus, a sequential approach based on both EHMRG and a consecutive echocardiographic assessment could be considered to improve the prognostic assessment in AHF without adding further examinations to the current suggested management. 

Bedside echocardiography is becoming widely adopted in emergency settings, currently performed by the attending physician in the ED in the first hours from patient’s arrival or by the cardiologist or internal medicine specialist in the first hours following the admission [21]. Bedside assessment also allows the non-cardiologist to perform an accurate differential diagnosis, an instrumental follow-up to assess treatment response, and a functional and prognostic appraisal [22,23]. This technique requires a specific training [24], and it is widely used in the ED to assess critical conditions as acute heart failure [23,25], pulmonary embolism [26,27,28], pericardial effusion [29,30], and shock states [31]. In the setting of AHF, bedside echocardiography allows medical staff to obtain reliable information regarding LVEF, right ventricle function, tricuspid regurgitant velocity, diastolic dysfunction, and atrial volumes [23]. Echocardiographic techniques are rapidly improving, and several newer methods have been suggested to improve the assessment of specific functions [32]. In our study, we chose to adopt TAPSE/PASp since this marker is strongly associated with AHF prognosis, and appears to be quite independent from left ventricle pathophysiology [33,34], which can be difficultly assessed in an emergency setting and in the first hours of admission. In fact, transthoracic LVEF evaluation can be affected by several physiological, technical, and clinical issues [35], and the described inter- and intra- observer variability of LVEF could imply a misclassification of the subjects [36]. Right-sided parameters, such as TAPSE and PASp, show a good intra-observer and inter-observer reliability [37], and have been directly associated to the degree of lung congestion, to a reduced decongestion after diuretic treatment, to increased natriuretic peptides at the admission [38], and to a globally worse prognosis in AHF, independently of LVEF [33].

Our study confirmed literature data, showing that both EHMRG and TAPSE/PASp predicted short-term prognosis in AHF adequately [11,15,39]. The most remarkable aspect of both tests was a good negative predictive value, thus being able to identify patients who will not undergo to in-hospital death. According to these properties, we observed a low risk of in-hospital death in EHMRG categories 1–3, similar to the one described in the original cohort [11]. Performing a bedside echocardiography in low-risk categories is still necessary for a differential diagnosis with other causes of dyspnea, and to guide pharmacological and non-pharmacological interventions, but little is added to EHMRG prediction; these patients showed an overall survival of 97.8% (89 subjects, two patients experiencing in-hospital death). 

Patients in Classes from 4 to 5b showed an overall lower survival rate, 87.7% (350 subjects, with 43 in-hospital deaths), but the prediction of short-term risk improved with the use of a sequential evaluation of TAPSE/PASp after EHMRG. In fact, considering the subgroup of patients submitted to bedside echography, we observed that a normal TAPSE/PAPs was able to identify patients with a lower risk of in-hospital death. In EHMRG Class 4 (64 subjects), we observed an overall of four deaths, with zero deaths in the normal TAPSE/PASp subgroup. In EHMRG Class 5a (54 subjects), we observed five deaths, with no cases in the normal TAPSE/PASp subgroup. Lastly, in EHMRG Class 5b (106 patients), we observed 34 deaths, only three of which occurred in the normal TAPSE/PASp group. 

Thus, subjects assessed to the ED for a diagnosis of AHF had a low-risk of short-term mortality if in EHMRG categories 1 to 3. Patients in high-risk categories (from EHMRG category 4 to 5b), if further assessed with TAPSE/PASp, showed a low risk of short-term events, similar to the one of lower-risk categories, if TAPSE/PASp was normal. A clinical, laboratoristic, and echocardiographic method could help the physician improving the prognostic assessment in elderly AHF subjects, as well as in other settings, such as pulmonary embolism [26]. 

Prediction of short-term survival is a key element in the management of AHF for both the ED physician and the internal medicine or cardiology specialist. A good prognostic pathway could be helpful guiding ED physician’s dispositions to admit or discharge safely and allowing resources optimization. Moreover, while cardiologists have already optimized and validated for their environment several prognostic scales for AHF, as ADHERE and OPTIMIZE-HF [40,41], internal medicine specialists lack of scores specifically validated for their populations that are often older and burdened by several comorbidities, and differ substantially from cardiology inpatients [42]. This issue has already been observed in other pathologies, as for example in subjects with sepsis [43], as well as subjects with pre-existing atrial fibrillation [44,45] admitted to Internal Medicine departments. EHMRG contains several items that are currently adopted in the prognostication of AHF and other critical illnesses in internal medicine, such as age, systolic blood pressure, creatinine, electrolyte imbalances, and troponin increase. These markers have a defined prognostic role also in other critical illnesses, often managed outside a cardiologic departments, such as sepsis and septic shock [46,47,48,49,50], pulmonary embolism [51], and pneumonia [52,53,54,55]. Thus, the adoption of these items in a predictive score seems more useful for an older, more complex, and less selected population, as the one commonly observed in the ED and in Internal Medicine. Of note, several papers underlined the modifications of the heart in specific comorbid conditions, such as chronic kidney disease [56], suggesting morphological differences that should also be taken into account when assessing a patient in Internal Medicine.

The current study has several limitations. Its single-center, retrospective design only allows suggesting hypotheses that should be validated in larger, multi-center, and prospective studies. To reduce bias, it would be interesting to assess survival in all the patients admitted to ED and then discharged. In fact, the current population analyzes only admitted subjects, and this could interfere with the generalizability of our results. Moreover, the reduced number of subjects in each subgroup strongly advocates larger numbers to confirm our observations.

## 5. Conclusions

EHMRG score is useful also in elderly patients admitted to Internal Medicine from the ED to assess AHF prognosis, and could be used to optimize patients’ management, thus could be useful to identify those at a lower risk of short-term death. A sequential assessment with bedside ultrasound adopting TAPSE/PASp could extend the EHMRG capacity to identify a lower risk of short-term death even among patients classified at a higher risk of death, thus allowing the physician to optimize the resource management and patients’ allocation.

## Figures and Tables

**Table 1:** EHMRG Score calculation.

Variable	Units	Factor
Age	years	2 × Age
ED arrival by ambulance	If “yes”	+60
SBP	mmHg	−1 × SBP
Heart Rate	beats/min	1 × heart rate
Oxygen Saturation	%	−2 × Oxygen Saturation
Creatinine	mg/dL	20 × Creatinine
Potassium	4.0–4.5 mmol/L ≥4.6 mmol/L ≤3.9 mmol/L	0 +30 +5
Troponin	> ULN	+60
Active cancer	If “yes”	+45
Metolazone at home	If “yes”	+60
Adjustment factor		+12
Total		EHMRG Score

Legend: EHMRG = Emergency Heart Failure Mortality Risk Grade; ED = emergency department; SBP = systolic blood pressure; ULN = upper limit of normal.

**Table 2:** Baseline characteristics of the sample.

Clinical Variables	All Sample (*n* = 439)
Age, years, (±SD)	84.6 (±7.7)
Males (n, %)	180 (41.0%)
In-hospital death (n, %)	45 (10.3%)
NYHA class, [IQR]	4 [1]
Length of hospitalization, days, [IQR]	10 [7]
BNP on admission, pg/mL, [IQR]	600.5 [805]
SBP, mmHg, (±SD)	127.5 (±28.1)
HR, bpm, (±SD)	89.4 (±24.6)
SpO2, %, (±SD)	91.8 (±7.3)
Creatinine, mg/dl, (±SD)	1.6 (±1.0)
Potassium, mmol/L, (±SD)	4.00 (±0.69)
Out of range Potassium, (n, %)	180 (41.1%)
Mean Troponin, ng/mL, [IQR]	0.05 [0.10]
Increased Troponin, (n, %)	204 (46.5%)
ED arrival by ambulance, (n, %)	284 (64.7%)
Active cancer, (n, %)	77 (17.9%)
Metolazone use, (n, %)	11 (2.6%)
EHMRG, [IQR]	69 [98.4]
EHMRG Class, [IQR]	5 [2]
**AHF Characteristics**	
ADHF (n, %)	370 (84.2%)
AHF de novo (n, %) Arrhythmia Hypertensive Crisis Other	36 (8.20%) 21 (4.78%) 12 (2.73%)
**Echocardiographic Variables**	**Valid Echocardiography (*n* = 289)**
Preserved EF, (n, %)	172 (59.5%)
TAPSE, mm, (±SD)	16.3 (±4.85)
PASp, mmHg, (±SD)	42.1 (±13.6)
E/e’, (±SD)	12.3 (±9.78)
E/A, [IQR]	1.00 [1.1]
LA Volume, ml/m^2^, (±SD)	64.9 (±24.0)
TAPSE/PASp, mm/mmHg, (±SD)	0.435 (±0.211)

Legend: BNP = brain-derived natriuretic peptide; EHMRG = Emergency Heart Failure Mortality Risk Grade; HR = heart rate; HFpEF = heart failure at preserved ejection fraction; IQR = interquartile range; LA = left atrium; SBP = systolic blood pressure; NYHA = New York Heart Academy; PASp = echocardiographically estimated pulmonary pressure; SD = standard deviation; SpO2 = oxygen saturation; TAPSE = tricuspid annulus plane systolic excursion.

**Table 3:** Cox regression analysis results.

	*p*	HR	95% Confidence Interval	
			Lower	Upper
EHMRG Category	0.01	2.206	1.180834	4.120832
TAPSE/PASp	0.001	37.69	4.09086	347.1741
NYHA Class	0.007	2.997	1.337784	6.712366
BNP at admission	0.874	1.000	0.999588	1.000484
LA Volume	0.012	1.031	1.006669	1.055831
LVEF	0.103	2.697	0.817866	8.894701

Legend: BNP = brain-derived natriuretic peptide; EHMRG = Emergency Heart Failure Mortality Risk Grade; HR = hazard ratio LA = left atrium; LVEF = Left Ventricle Ejection Fraction; NYHA = New York Heart Academy; PASp = echocardiographically estimated pulmonary pressure; TAPSE = tricuspid annulus plane systolic excursion.

## Data Availability

The study data are available upon reasonable request.
